# Trends in H_2_S-Donors Chemistry and Their Effects in Cardiovascular Diseases

**DOI:** 10.3390/antiox10030429

**Published:** 2021-03-11

**Authors:** Angela Corvino, Francesco Frecentese, Elisa Magli, Elisa Perissutti, Vincenzo Santagada, Antonia Scognamiglio, Giuseppe Caliendo, Ferdinando Fiorino, Beatrice Severino

**Affiliations:** Department of Pharmacy, School of Medicine, University of Naples Federico II, Via D. Montesano, 49, 80131 Napoli, Italy; angela.corvino@unina.it (A.C.); frecente@unina.it (F.F.); elisa.magli@unina.it (E.M.); perissut@unina.it (E.P.); santagad@unina.it (V.S.); antonia.scognamiglio@unina.it (A.S.); caliendo@unina.it (G.C.); fefiorin@unina.it (F.F.)

**Keywords:** hydrogen sulfide, natural H_2_S donors, synthetic H_2_S donors, triggered mechanism, H_2_S release, cardiovascular diseases

## Abstract

Hydrogen sulfide (H_2_S) is an endogenous gasotransmitter recently emerged as an important regulatory mediator of numerous human cell functions in health and in disease. In fact, much evidence has suggested that hydrogen sulfide plays a significant role in many physio-pathological processes, such as inflammation, oxidation, neurophysiology, ion channels regulation, cardiovascular protection, endocrine regulation, and tumor progression. Considering the plethora of physiological effects of this gasotransmitter, the protective role of H_2_S donors in different disease models has been extensively studied. Based on the growing interest in H_2_S-releasing compounds and their importance as tools for biological and pharmacological studies, this review is an exploration of currently available H_2_S donors, classifying them by the H_2_S-releasing-triggered mechanism and highlighting those potentially useful as promising drugs in the treatment of cardiovascular diseases.

## 1. Introduction

Hydrogen sulfide (H_2_S), the third endogenous recognized gaseous signaling transmitter [1], among nitric oxide (NO) and carbon monoxide (CO), is a colorless and pungent gas with a boiling point of 60 °C [2].

It is endogenously synthesized by four enzymes: cystathionine γ-lyase (CSE) and cystathionine β-synthetase (CBS), which catalyze the production of H_2_S by a direct enzymatic desulfhydration of L-cysteine, and 3-mercaptopyruvate sulfurtransferase (3-MST), which produces H_2_S by an indirect desulfhydration, in concert with cysteine aminotransferase (CAT) and in the presence of reductants [3]. The expression of these enzymes is tissue specific. The amount of CBS is found mostly in the central nervous system, the liver, the kidney, the uterus, and placenta; CSE is primarily concentrated in the cardiovascular system, whereas 3-MST is located predominantly in the mitochondria [4,5,6,7,8].

To study the biological effects of endogenously synthesized H_2_S, the development of inhibitors selective for the above enzymes is required. To date, some selective inhibitors of CSE and CBS have been identified and widely used in biological systems [9,10,11,12,13]; conversely, no pharmacological inhibitors of the enzyme 3-MST have yet been discovered.

Several studies have shown that H_2_S plays a significant modulatory role in numerous physio-pathological processes in the human body [14]. In fact, it is involved in the homeostatic regulation of respiratory, cardiovascular, immune, nervous, gastroenteric, and endocrine systems [4,15].

**Figure 1:**
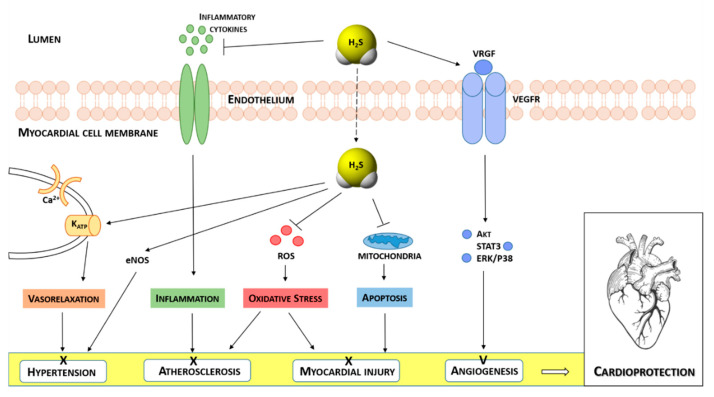
Schematic illustration of the effects of H_2_S in different heart diseases and the molecular mechanisms underlying H_2_S-induced cardioprotection.

Currently, growing research evidence has confirmed the protective effects of H_2_S in cardiovascular diseases [16,17,18,19,20,21,22,23], such as cardiac hypertrophy, heart failure, myocardial ischemia/reperfusion (I/R) injury [24], hypertension [18,25] and atherosclerosis [26], acting as an activator of angiogenesis [27], basal vasorelaxant agent [28], blood pressure, and heart rate regulator [29,30]. Moreover, it has been demonstrated that the related mechanisms of action accounting for this cardioprotective activity involve antioxidation, inhibition of cell apoptosis, pro-angiogenesis, anti-inflammatory, and ion channels regulation [31,32] (Figure 1).

As depicted in Figure 1, H_2_S exerts a cardioprotective effect by activating different endothelium-dependent signaling pathways [33]. It reduces blood pressure, by inducing vasorelaxation, a consequence of opening K_ATP_ channels and increasing K^+^ currents resulting in hyperpolarizing membrane of smooth muscle cells [34]. Moreover, H_2_S shows its anti-hypertensive effect, by activating endothelial nitric oxide synthase (eNOS) and increasing NO bioavailability [35]. It also shows an inhibitory effect on the pathogenesis of atherosclerosis, by preventing an inflammatory response mediated by inflammatory cytokines [26] and exerting antioxidative action through the activation of the nuclear factor erythroid 2-related factor 2 (Nrf2)-dependent pathway and the protection of tissues by reactive oxygen species (ROS). The cardioprotection of H_2_S is also associated with the inhibition of cardiomyocyte apoptosis after myocardial injury. In fact, it suppresses the activation of caspase-3 and upregulates the expression of glycogen synthase kinase-3 (GSK-3*β*). The antioxidant effect of H_2_S is also embodied in the preservation of mitochondrial functions by inhibiting mitochondrial respiration [36].

Furthermore, H_2_S stimulates angiogenesis by increasing the expression of VEGF and activating some downstream effectors, such as Akt, STAT3, ERK, and p38 [37].

Recently, considering the interesting involvement of H_2_S in the cardiovascular system, there has been heightened enthusiasm for the development of compounds able to generate exogenous H_2_S, which could represent biological tools and promising cardioprotective agents.

## 2. H_2_S Donors

For the past several years, inorganic sulfide salts, such as sodium sulfide (Na_2_S) and hydrosulfide (NaHS), have been commonly used as pharmacological tools. They represent the early H_2_S donors used in biomedicine field, the results of which make it very useful to elucidate the physio-pathological roles of H_2_S in mammalian systems.

Although these sulfide salts have been the basic tools used for H_2_S research for years, some of their significant limitations were demonstrated. In fact, sulfide salts hydrolyze immediately upon reaction with water and the resulting too-rapid release of H_2_S causes its blood and tissue concentrations to surge to supraphysiological levels followed by a rapid drop [38].

Therefore, this suboptimal pharmacokinetic profile led the researchers to limit the use of these salts as potential therapeutics and to obtain novel organic H_2_S-releasing compounds, including chemically synthesized molecules and natural plant extracts. 

One of the first slow-releasing H_2_S donors was Lawesson’s reagent, a synthetic compound which is largely used for sulfurization of organic molecules [39]. It has been used in some pharmacological studies as an H_2_S donor, despite its lack of water solubility. 

Therefore, to improve this chemical property, GYY4137, a water-soluble derivative of Lawesson’s reagent, was obtained. Similarly to its parent compound, it also releases H_2_S upon hydrolysis, but it better mimics the physiological H_2_S production, due to its slow-releasing nature [40,41]. Moreover, in order to optimize the H_2_S release properties of GYY4137, structural modifications were made to this compound via substitution of the P-C bond in GYY4137 with a P-O bond, affording a series of O-substituted phosphorodithioate-based H_2_S donors [42].

Later on, naturally occurring H_2_S donors derived from some vegetables belonging to Alliaceae and Brassicaceae, such as garlic, onion, broccoli, cabbage, watercress, and garden cress, were also investigated. 

So far, among garlic-derived compounds, allicin is one of the best characterized. Due to its instability in aqueous media, it quickly decomposes into four H_2_S-releasing compounds: diallyl sulfide (DAS), diallyl disulfide (DADS), diallyl trisulfide (DATS), and ajoene [43].

Other garlic-derived compounds containing a sulfur atom are S-allylcysteine (SAC) with its structural analogs, S-propyl-L-cysteine (SPC), and S-propargyl-cysteine (SPRC), which are potential sources of H_2_S [44,45,46].

Similarly, some cruciferous vegetables contain natural isothiocyanates, such as sulforaphane, allyl isothiocyanate, benzyl isothiocyanate, 4-hydroxybenzyl isothiocyanate, and erucin, which exhibit a significant H_2_S-releasing activity [47].

Moreover, it has been demonstrated that some thioamino acids, such as thioglycine and thiovaline, are also H_2_S donors, showing a slow release of the gasotransmitter [48].

Recently, the limited clinical application of some naturally occurring H_2_S donors led to the development of novel synthetic compounds with favorable pharmacological properties, which could be useful both as research tools and as potential therapeutics.

Thus, in order to obtain effective H_2_S donors with controllable release rates, a series of N-(benzoylthio)benzamides [49], acyl perthiols [50,51], arylthioamides [52], 1,2,4-thiadiazolidin-3,5-diones [53], iminothioethers [54], mercaptopyruvate [55], dithioates [56], isothiocyanate [57,58], and thiocarbamates [59] were developed and evaluated for their H_2_S-releasing properties. 

Each of these donors release H_2_S with different mechanisms, triggered by hydrolysis, pH modulation, cellular thiols, photo activation, enzymatic reaction, or others.

### 2.1. Sulfide Salts

Over the years, sulfide salts, such as sodium sulfide (Na_2_S) and hydrosulfide (NaHS), have been widely used in biological studies to explore the therapeutic prospects of exogenous source of H_2_S. In particular, these salts showed protective effects against many disease states, such as inflammation [60,61], acute lung injury [62,63], oxidative stress in human neuroblastoma cells [64], Alzheimer’s disease (AD) [65], atherosclerosis [66], and ulcer [67,68].

In addition to these effects, aqueous NaSH solutions delivered in aortic rings led to a 60% greater relaxation over controls, confirming the vasorelaxant properties of H_2_S [28].

Moreover, several groups demonstrated that the use of sulfide salts was able to induce either pre- and post-conditioning cardioprotection [25,69,70,71,72,73]. 

Exogenous NaHS has proven to be useful in attenuating ischemia-reperfusion injury [74], protecting against myocardial infarction (MI) [75] and hemorrhagic shock [76].

Furthermore, Calvert et al. reported that long-term Na_2_S administration attenuates ischemia-induced heart failure, by reducing oxidative stress [24]. The results suggested that the H_2_S therapy attenuates left ventricular (LV) dilation and cardiac hypertrophy, and leads to an improvement in cardiac function. 

Solutions of these sulfide salts are prepared by using hydrates of sodium hydrogen sulfide (NaHS × nH_2_O), nonahydrate disodium salt (Na_2_S × 9 H_2_O), or the anhydrous form.

**Figure 2:**
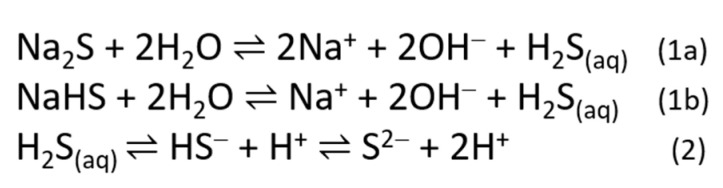
Mechanism of H_2_S release from Na_2_S (**1a**) and NaHS (**1b**) in aqueous solution and its dynamic equilibrium among different species (**2**).

In water or aqueous solution, these sulfide salts quickly hydrolyze, establishing a dynamic equilibrium among sulfide ions (S^2−^), bisulfide ions (HS^−^), and molecular hydrogen sulfide (H_2_S) [77] (Figure 2); the ratio of these species depends on different parameters, such as temperature, pressure, and pH. 

As the pH increases, the H_2_S dissociates into its ions. At a pH below 6, H_2_S is the predominant sulfur species; at the physiological pH, H_2_S and HS^−^ species are both present in solution in the ratio of 1:3; at a pH of ~9 HS^−^ is fully formed, while at a pH above 15, S^2−^ is the predominant species.

### 2.2. Naturally Occurring Donors

Currently available H_2_S-releasing compounds can be divided into two groups: naturally occurring donors and synthetic donors. Among the natural source, allium family and cruciferous vegetables are recognized to be rich in organosulfur compounds.

In particular, garlic contains at least thirty-three sulfur compounds, a concentration higher than any other allium species. The organosulfides in the allium family are represented by oil-soluble polysulfides and water-soluble thiosulfinates and appear to be responsible for many garlic’s medicinal effects. It has been demonstrated that the allicin, one of the biologically active components in garlic, and its principal transformation products, which are organic polysulfides, provide a critical role in garlic-induced cardioprotection [78,79,80,81].

In fact, it has been demonstrated that these compounds are responsible for lowering the arterial blood pressure, reducing the serum cholesterol and triglycerides, inhibiting the platelet aggregation, preventing the atherosclerosis, increasing the fibrinolytic activity, and reducing the oxidative stress [82].

**Figure 3:**
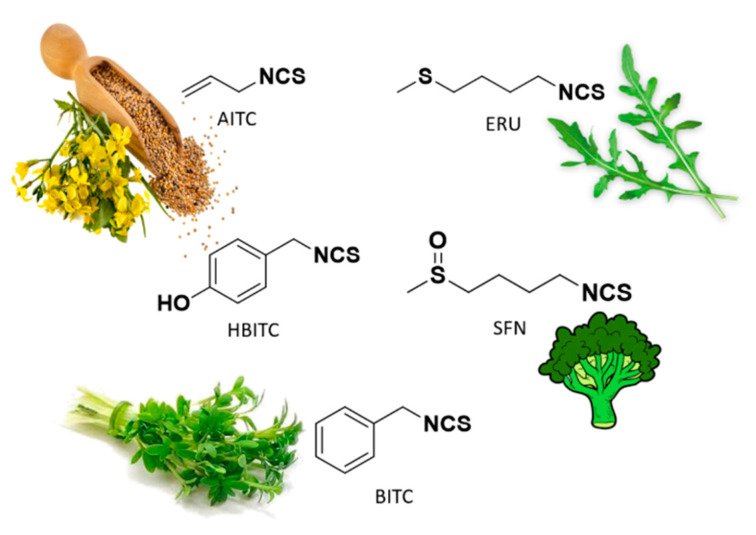
Chemical structures of natural isothiocyanates, which are abundant in cruciferous vegetables.

Among Cruciferae, vegetables such as broccoli, watercress, mustard, and garden cress are rich in isothiocyanates, such as sulforaphane (SFN, highly present in broccoli), allyl isothiocyanate (AITC, highly present in black mustard), benzyl isothiocyanate (BITC, highly present in garden cress), 4-hydroxybenzyl isothiocyanate (HBITC, highly present in white mustard), and erucin (ERU, mainly present in broccoli and rocket) (Figure 3).

Recently, it has been demonstrated that these secondary metabolites of Brassicaceae, derived from corresponding glucosinolates upon the enzymatic action of myrosinase, show H_2_S-releasing properties. This finding shed light on the mechanism of action of Brassicaceae and on the role of hydrogen sulfide as relevant player for their nutraceutical and pharmaceutical effects [47]. 

Based on the mechanism of release, the above compounds belong to thiol-triggered donors; in fact, they react with the thiol groups of glutathione (GSH) or cysteine to release free H_2_S. 

In the intact garlic, the primary sulfur compounds are the γ-glutamyl-S-alk(en)yl-L-cysteines which can be hydrolyzed and oxidized to yield alliin. Alliinase accounts for the conversion of alliin to allicin. The latter is highly instable in aqueous media and instantly decomposes, forming some oil-soluble compounds, such as diallyl sulfide (DAS), diallyl disulfide (DADS), diallyl trisulfide (DATS), and ajoene [43]. These allicin-derived compounds led to H_2_S generation upon reaction with endogenous thiols, such as L-cysteine and GSH.

**Figure 4:**
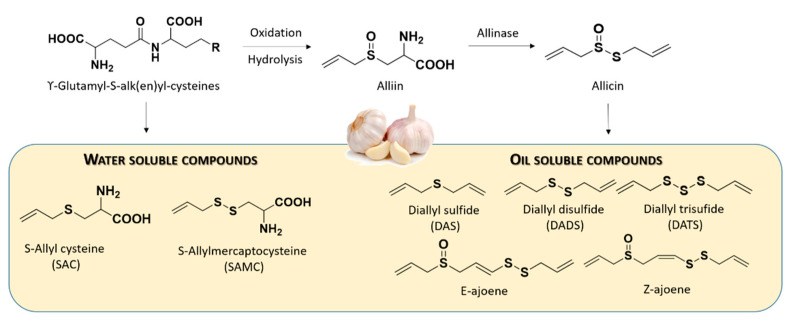
Chemical structures of commonly studied organosulfur compounds of garlic.

Furthermore, γ-glutamyl-S-alk(en)yl-L-cysteines can be also converted to water-soluble organosulfur compounds including S-allyl cysteine (SAC) and S-allyl mercaptocysteine (SAMC) (Figure 4).

SAC, a sulfur-containing amino acid, was considered an endogenous H_2_S-producing agent by providing the substrate for CSE in the H_2_S synthesis (Figure S1).

It is responsible for many important activities, such as antioxidative [83,84], anticancer [85,86], anti-hepatotoxic [87,88], and cardioprotective activities [89]. In particular, it showed a cardiovascular protective role in MI by decreasing lipid peroxide products and improving the antioxidant status. Indeed, Chuah et al. also demonstrated that SAC could upregulate the expression of CSE, leading to enhanced H_2_S release. The increased H_2_S concentration in the myocardial and plasma is responsible for cardioprotection during acute MI [90]. 

Moreover, it was reported that S-propargyl-cysteine (SPRC) and S-propyl-cysteine (SPC) (Figure S1) also exhibited cardioprotective effects, via CSE/H_2_S pathway [91,92].

**Figure 5:**
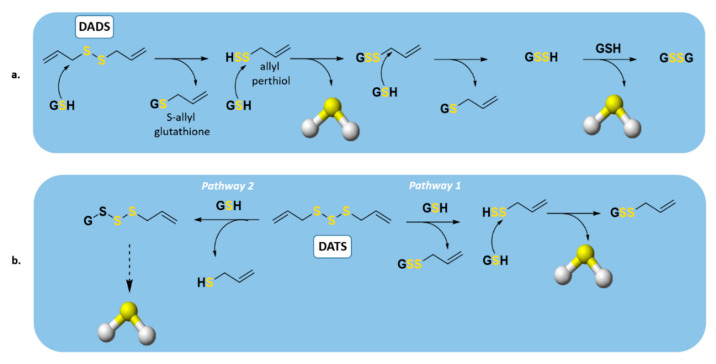
H_2_S production from organic polysulfides by thiol reactions. Proposed mechanism of H_2_S production from (**a**) diallyl disulfide (DADS) and (**b**) diallyl trisulfide (DATS).

Among the oil-soluble components of garlic, the major organosulfur compounds were reported to be DADS and DATS. In 2007, Benavides et al. demonstrated that these compounds could release H_2_S rapidly through a thiol-dependent mechanism [93] (Figure 5). In the presence of GSH, DADS can react with it to generate S-allyl glutathione (GSA) and the intermediate allyl perthiol (ASSH), which reacts with another GSH to produce H_2_S and S-allyl glutathione disulfide (GSSA). Furthermore, the latter compound could also undergo α-carbon nucleophilic substitution, generating H_2_S rapidly [78] (Figure 5a). This mechanism has been widely confirmed [33,94,95,96,97,98,99], except for the rate of H_2_S release from DADS. Regarding this topic, in fact, Liang and co-workers demonstrated that DADS releases H_2_S very slowly through α-carbon nucleophilic substitution. The explanation of the previous misunderstanding of DADS as a fast H_2_S donor is DATS contamination in commercial samples of DADS; in fact, among these compounds, DATS is responsible for the rapid H_2_S release.

Regarding DATS (Figure 5b), two possible thiol−disulfide exchange reaction pathways can occur. Firstly (pathway 1), the nucleophilic attack of GSH on allylic sulfur of DATS can generate GSSA and ASSH, with the latter able to be reduced by GSH and thus releasing H_2_S; on the other side (pathway 2), the nucleophilic attack of GSH on the central sulfur atom of DATS can lead to the production of ASH and GSSSA. The latter can generate H_2_S rapidly. 

**Figure 6:**
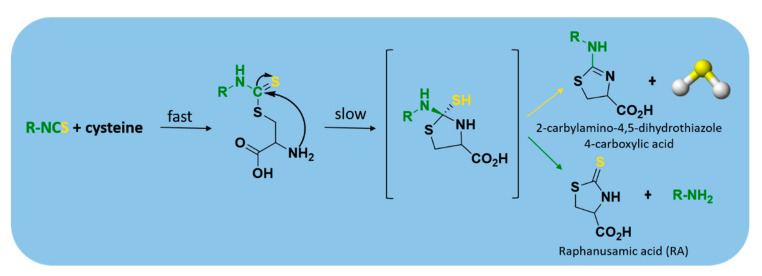
The mechanism of H_2_S release from isothiocyanates.

Currently, isothiocyanates are also drawing a lot of attention for their many biological and pharmacological effects in the prevention of important human diseases, such as cancer, neurodegenerative processes, and cardiovascular diseases [100]. In particular, Martelli et al. demonstrated that some selected aryl isothiocyanates were able to release H_2_S in a biological environment and produce a vasorelaxant effect in rat aortic rings, strongly antagonized by a specific Kv7-blocker, and in the coronary vascular bed, causing an increase of basal coronary flow [57]. Furthermore, the H_2_S-releasing properties of the secondary metabolites of Brassicaceae, isothiocyanates, are also derived from the rapid reaction with thiols, such as cysteine (Figure 6).

The formed Cys-ITC adduct undergo intramolecular cyclization followed by releasing organic amine R−NH_2_ and raphanusamic acid (RA), on one hand, and H_2_S and 2-carbylamino-4,5-dihydrothiazole-4-carboxylic acid, on the other hand [101].

### 2.3. Synthetic Donors

Although naturally occurring H_2_S donors represent an attractive tool for in vivo studies, their poor toxicity and the tendency of these compounds to also generate some byproducts, which are not related to H_2_S production, led researchers to develop novel synthetic H_2_S donors with more favorable pharmacokinetic and safety profiles. 

Based on their mechanism of H_2_S release, we have classified the synthetic compounds into different groups: hydrolysis-triggered donors, pH-controllable H_2_S donors, thiol-triggered donors, enzyme-triggered donors, and others (Table 1).

#### 2.3.1. Hydrolysis-Triggered Donors

Morpholin-4-ium-4-methoxyphenyl(morpholino)phosphinodithioate (GYY4137), a water-soluble Lawesson’s reagent derivative (Figure S2), is one of the first slow-releasing H_2_S donors developed [40,41,102] and the most commonly used research tool to investigate the role of H_2_S in the biological systems.

GYY4137 was synthesized starting from Lawesson’s reagent, previously obtained by heating a mixture of anisole with phosphorus pentasulfide (P4S10) [103,104], which reacts with morpholine in dichloromethane at room temperature (Figure S3) [40].

Much evidence has revealed that GYY4137 exhibits anti-inflammatory, antioxidant, and anticancer properties [52].

It also activates vascular smooth muscle K_ATP_ channels, and relaxes rat aortic rings and renal blood vessels, showing its anti-hypertensive activity [40].

Moreover, GYY4137 has been reported to inhibit microvascular thrombus formation and to stabilize atherosclerosis plaque by interfering with platelet activation and adhesion molecule-mediated aggregation [105]. It also protects against diabetic myocardial I/R injury, through activation of the PHLPP-1/Akt/Nrf2 pathway [106].

It has been demonstrated that GYY4137 slowly releases H_2_S upon hydrolysis [40]. In 2015, Alexander et al. carefully studied the hydrolysis kinetics of GYY4137, monitoring it by a combination of NMR spectroscopy and mass spectrometry [107]. Firstly, a sulfur–oxygen exchange with water occurs, leading to the release of H_2_S. The formed product, an aryl-phosphonamidothioate, undergoes complete hydrolysis to release a second molecule of H_2_S (Figure S4).

Despite GYY4137 having been proven to be a useful biological tool, it suffers from some drawbacks, such as its contamination with traces of dichloromethane residual from crystallization and the slow hydrolysis rate. These aspects could make the attribution of biological effects to GYY4137-derived H_2_S uncertain, because of the possible simultaneous metabolization of dichloromethane to CO, which has biological effects like H_2_S [108]. 

Therefore, structural modifications of GYY4137 were designed and the resulting analogs were studied. Park and coworkers developed a series of O-aryl- and alkyl-substituted phosphorodithioates as H_2_S donors, by replacing the P-C bond in GYY4137 with O-substitution [42] (Figure S2). Their studies evidenced that the gaseous release from these novel H_2_S donors did not significantly improve. In fact, similarly to the parent compound GYY4137, O-arylated donors showed slow H_2_S production, whereas O-alkylated donors exhibited very weak H_2_S generation.

Another class of compounds belonging to the family of hydrolysis-triggered H_2_S donors are 1,2-dithiole-3-thiones (DTTs) (Figure S2).

DTT compounds are anethole dithiolethione (ADT) and its O-demethylated derivative ADT-OH [5-(*p*-hydroxyphenyl)-3H-1,2-dithiole-3-thione], which were largely used as H_2_S donors (Figure S5).

To obtain DTTs, different methods can be applied. The most used synthetic strategy provides dehydrogenation and sulfurization of an allylic methyl group, by treating it with elemental sulfur or phosphorus pentasulfide products [109] (Figure S6). Alternatively, β-ketoesters could react with Lawesson’s reagent to give the desired DTTs [110,111] (Figure S6).

To date, several groups have studied the biological effects of ADT and ADT-OH, observing an important activity related to the H_2_S-releasing properties of these compounds. ADT is an FDA-approved drug, which can stimulate bile secretion, restoring salivation and relieving dry mouth in patients affected by chemotherapy-induced xerostomia [112]. Additionally, its derivative, ADT-OH, resulted in being useful for reducing cell viability via inhibition of histone deacetylase [113,114] and NF-kB activation [115].

Although their H_2_S-releasing mechanism is still not completely defined, it is widely accepted that the production of H_2_S from DTTs occurs via hydrolysis [34,116,117] (Figure S7). Indeed, it has been demonstrated that upon heating to 120 °C in aqueous solution, dithiolethione derivatives gradually release H_2_S, converting into the corresponding 1,2-dithiole-3-one [39].

Interestingly, H_2_S donor hybrids were obtained by coupling the OH group of ADT-OH with some commercially available drugs and the resulting compounds were studied for their H_2_S-releasing properties and therapeutic effects [118].

**Figure 7:**
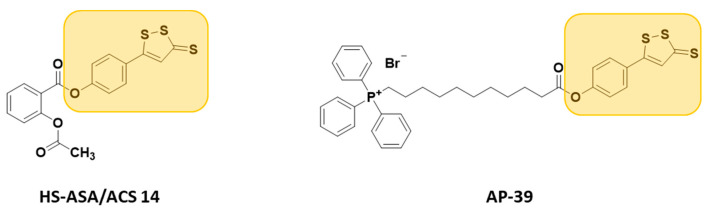
Chemical structures of H_2_S donor hybrids involved in cardiovascular disease.

They are usually synthesized by coupling the active compounds with ADT-OH in the presence of N,N’-dicyclohexylcarbodiimide (DCC) and 4-dimethylaminopyridine (DMAP) (Figure S8) [119]. These H_2_S donor hybrids exhibited their efficacy in many pharmacological fields, such as inflammation [120,121] (H_2_S-donating diclofenac, ACS-15/ATB-337; H_2_S-donating mesalamine, ATB-429), cancer [122] (H_2_S-donating sulindac, HS-SUL; H_2_S-donating aspirin, HS-ASA/ACS-14; H_2_S-donating ibuprofen, HS-IBU; H_2_S-donating naproxen, HS-NAP), erectile dysfunction [123] (H_2_S-donating sildenafil, ACS 6), neurodegeneration (H_2_S-donating latanoprost, ACS 67, for glaucoma treatment [124], and H_2_S-donating levodopa, ACS 83, for Parkinson’s disease [125]), and cardioprotection (H_2_S-donating aspirin, HS-ASA/ACS 14, and AP-39) (Figure 7).

Indeed, Rossoni and co-workers demonstrated that ACS14 exerts strong protective effects against buthionine sulfoximine (BSO)-induced cardiovascular diseases, by increasing systolic blood pressure and lowering heart rates in rats [126]. Additionally, oral administration of ACS 14 reduces BSO-induced hypertensive effects, unlike aspirin which has no effect. Moreover, ACS 14 treatment in BSO rats reduces myocardial I/R injury [126,127,128].

More recently, another hybrid H_2_S donor, named AP-39, resulting from the reaction of triphenylphosphonium with ADT-OH, was developed [129] and studied for its H_2_S-releasing properties and potential pharmacological effects. 

AP-39 significantly inhibits oxidative stress-induced toxicity and protects against acute cardiac arrest, renal, and myocardial I/R injury, by inhibiting the mitochondrial permeability transition pore [130,131,132,133].

#### 2.3.2. pH-Controllable H_2_S Donors 

Starting from GYY4137 structure, Kang and coworkers developed a series of phosphonamidothioates, named JK donors [134]. 

To synthesize these compounds, the phenylphosphonothioic dichloride was treated with 3-hydroxypropionitrile and, consequently, with a selected C-protected amino acid, such as glycine, phenylalanine, valine, alanine, and proline. These steps provided the intermediate, which underwent LiOH-mediated hydrolysis, furnishing the final compounds (Figure S9).

These donors represent the first class of pH-controllable H_2_S donors. Indeed, in aqueous media under acidic conditions (pH = 2.0–6.0), JK donors had higher H_2_S release rates, whereas at neutral and mildly basic pH (7.4–8.0), they caused slower and less H_2_S release. 

**Figure 8:**
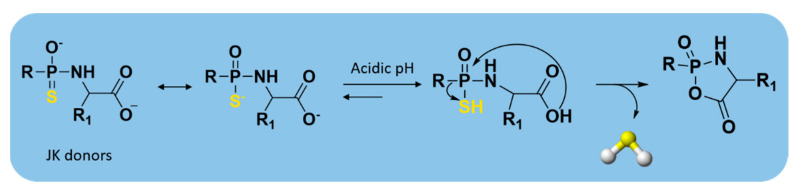
H_2_S-releasing mechanism from JK donors.

Kang and co-workers observed that these donors undergo a new hydrolysis mechanism of release. The protonation of phosphonamidothioates leads to the formation of the corresponding phosphorothiols, which cyclize via nucleophilic addition of the carboxylic acid group, resulting in breakage of the P-S bond and in release of H_2_S (Figure 8).

Moreover, the two main donors (JK-1 and JK-2) of the series (Figure S10) showed protective effects on cellular and murine models of myocardial I/R injury. They were also successful in reducing infarct size and circulating troponin-I level significantly [134].

Furthermore, ammonium tetrathiomolybdate (ATTM), an excellent copper chelator with the formula (NH_4_)_2_MoS_4_, was identified as a pH-dependent H_2_S donor [135]. Indeed, it has been demonstrated that H_2_S can be generated from ATTM under strong acidic conditions (5% H_2_SO_4_) (Figure S11).

Later, Xu and co-workers described ATTM as a water-soluble and a pH-sensitive slow-releasing H_2_S donor [136]. More recently, it has been found that ATTM exerts protective effects on the heart and brain in rat models of myocardial and cerebral I/R injury and attenuates I/R injury [137]. 

Considering that some preclinical data suggested that H_2_S is responsible for protective effects in doxorubicin- and adriamycin-induced cardiomyopathy during cancer therapies [138,139,140] and that ATTM is currently in clinical trials for the treatment of breast cancer due to its copper depletion effects [141], the researchers are studying the potential dual role of ATTM as an anticancer and cardioprotective agent in patients with cancer treated with doxorubicin or adriamycin.

#### 2.3.3. Thiol-Triggered Donors 

Thiol-activated H_2_S-releasing compounds were some of the first synthetic donors to be reported. They can release H_2_S by reacting with endogenous thiol-containing molecules, such as GSH, which are relatively abundant in mammals. Some of these H_2_S donors were found to exhibit protective effects in cardiovascular diseases (Figure S12).

Among the first nucleophile-triggered H_2_S donors, a series of N-(benzoylthio)benzamides were synthesized by Zhao et al. [49] with the goal of developing compounds which are stable in aqueous solution. The N-(acylthiol)benzamide derivatives were prepared by treating thiocarboxylic acids with hydroxylamine-O-sulfonic acid under basic conditions; the following reaction between the obtained S-acylthiohydroxylamines and benzoic anhydride led to obtain the final products (Figure S13).

The resulting compounds were proven to be stable in aqueous buffers and to be able to release H_2_S only following a nucleophilic attack by thiols. The structure–activity relationship studies showed that electron withdrawing groups cause a faster H_2_S release, unlike electron donating groups. In addition, H_2_S release from N-(benzoylthio)benzamides was also detected in the plasma, revealing that N-SH compounds have efficacy in complex systems. 

These compounds protect human keratinocytes against methylglyoxal (MGO)-induced cell damage and dysfunction, which is prevalent among patients with diabetes mellitus [142]. Moreover, they were evaluated in animal models of myocardial I/R injury. The reduction in infarct size over controls in murine models indicated N-(benzoylthio)benzamides as cardioprotective agents [143].

**Figure 9:**
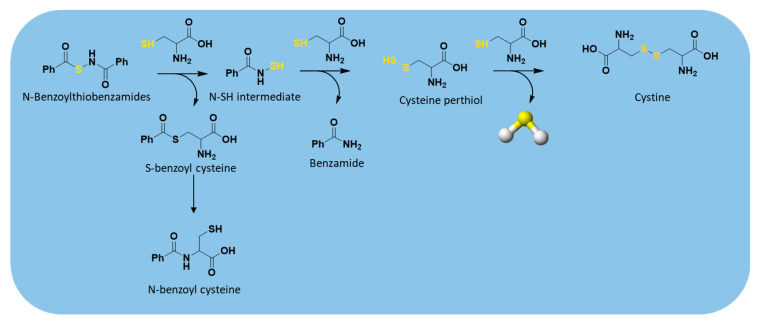
H_2_S release from N-benzoylthiobenzamides.

The thiol-triggered mechanism of release from these H_2_S donors starts with a thioester exchange between the donor and a first molecule of cysteine generating S-benzoyl cysteine and N-mercaptobenzamide, an N-SH intermediate (Figure 9).

The resulting S-benzoyl cysteine, due to its high reactivity, undergoes a fast S to N acyl transfer, leading to the formation of a more stable N-benzoyl cysteine. At the same time, the formed N-SH intermediate reacts with another molecule of cysteine to give benzamide and cysteine perthiol, which finally reacts with cysteine to generate cystine and release H_2_S.

As cysteine perthiol was found to be the key intermediate for H_2_S generation from N-mercapto-based donors, in addition to its already-known involvement in H_2_S biosynthesis catalyzed by CSE [118,144], a library of perthiol-based donors (RC(O)–S–SR’) were designed and developed by Zhao and co-workers [50], aiming to mimic H_2_S bioproduction.

The authors prepared the above compounds as cysteine and penicillamine derivatives (Figure S14) and protected the unstable –SH residue with acyl groups. 

Cys-S-SH-based donors were obtained through the treatment of N-benzoyl cysteine methyl ester with 2,2′-dipyridyl disulfide to attain a reactive intermediate, which then reacted with a thioacid to produce the desired compounds (Figure S15).

To obtain the other derivatives, C- and N-protected penicillamine was treated with 2,2′-dibenzothioazolyldisulfide, furnishing an intermediate, which quickly reacted with different thioacids to produce the desired donors (Figure S16).

Surprisingly, it was found that these donors exhibit different H_2_S-releasing profiles. Indeed, in the presence of thiols, penicillamine-based donors showed higher efficiency in H_2_S production than cysteine-based donors, which were able to release very small amounts of H_2_S. Probably, the two different behaviors could be explained by the presence of the two adjacent methyl groups of penicillamine-based donors, which prevent the cleavage of the disulfide bonds by thiols. 

These donors showed tunable release rates by varying the aromatic R substituent. H_2_S release depends on both steric and electronic factors, with electron-withdrawing substituents accelerating release rates, and bulky substituents on the aromatic ring retarding release rates. 

**Figure 10:**
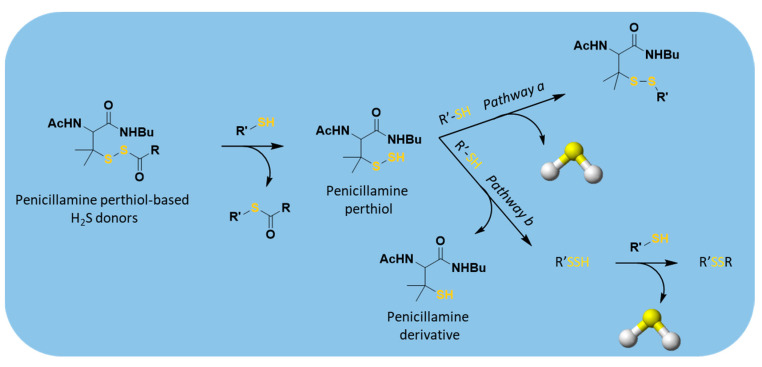
Proposed mechanism of H_2_S release from perthiol-based donors; R’-SH = cysteine or GSH.

Regarding the H_2_S-releasing mechanism, the acyl perthiol donors also required disulfide exchange to allow H_2_S production, similarly to N-mercapto-based donors; therefore, deacylation and subsequent H_2_S release occurred (Figure 10).

H_2_S release from penicillamine-derived perthiol donors was also studied in vivo and in cardiac myocytes (H9c2 cells), confirming their ability to release. Moreover, the selected donors were evaluated into a murine model of myocardial I/R injury. The results proved that they exhibit cardioprotective activity due to their ability to reduce the infarct size.

Analogous perthiol-based donors are the dithioperoxyanhydrides (RC(O)–S–SC(O)R’) [51], which also have the disulfide linkage in their structures (Figure S12). 

Both alkyl and aromatic dithioperoxyanhydride donors were obtained by two possible synthetic strategies: iodine oxidation of the thiocarboxylates [145] or reactions between thiocarboxylic acids and methoxycarbonylsulfenyl chloride [146] (Figure S17).

**Figure 11:**
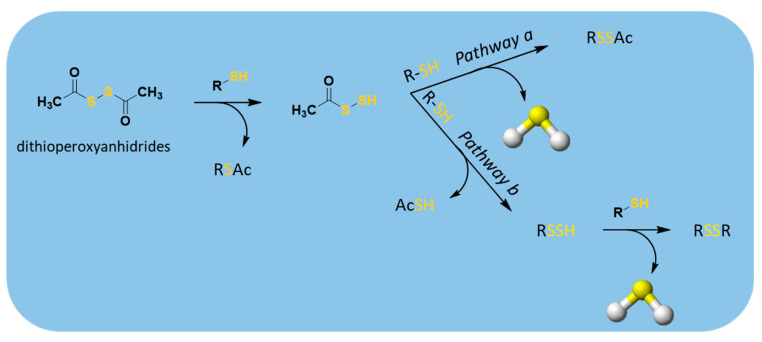
Proposed mechanism for H_2_S release from dithioperoxyanhydrides; R′-SH = cysteine or GSH.

H_2_S release from these compounds was measured amperometrically and found in both buffers and cellular lysates, confirming H_2_S-releasing properties of dithioperoxyanhydrides. When treated with thiols, these donors formed a key intermediate for H_2_S production, the acyl-persulfides, which, reacting with the excess of thiols, could directly release H_2_S and RSSAc (pathway a, Figure 11); alternatively, it could produce a new perthiol species (RS-SH), which reacting in turn with thiols, leads to disulfide formation and H_2_S release (pathway b, Figure 11).

Furthermore, these donors were found to induce concentration-dependent vasorelaxation of pre-contracted rat aortic rings, presumably owing to H_2_S release. Moreover, the arylthioamides (Figure S12) were also classified as thiol-activated H_2_S donors [52]. The non-heterocyclic aromatic compounds were obtained by mixing the corresponding benzonitrile with P_4_S_10_; whereas the reaction between amides with Lawesson’s reagent led to heterocyclic aromatic compounds being obtained (Figure S18).

These compounds showed the ability to release H_2_S in the presence of thiols, similarly to DADS and GYY4137, two slow-releasing donors. To date, the H_2_S release mechanism and relative intermediates and final products have not yet been clearly described.

After confirming their H_2_S-releasing profile, Calderone and coworkers tested the effects of these donors on noradrenaline-induced vasoconstriction in isolated rat aortic rings. The results suggested that the lead compound, *p*-hydroxybenzothioamide (Figure S19), has protective activity in cardiovascular systems, inhibiting norepinephrine-induced vasoconstriction in ex vivo in rat aortic rings, and reducing systolic blood pressure.

Considering the slow and sustained H_2_S release profile of *p*-hydroxybenzothioamide and its ease of conjugation to other compounds, this donor was used to develop many hybrid compounds. Among these, *p*-hydroxybenzothioamide was conjugated with the active compound naproxen, obtaining ATB-346 (Figure S19) [147]. Thus, the efficacy of this hybrid as an anticancer drug was investigated, confirming its apoptotic action in human melanoma cells [148]. Moreover, it has been demonstrated that ATB-346 reduces gastrointestinal tract injury and exhibits chemopreventive action against colorectal cancer when compared to naproxen [149]. Recently, a phase II study was completed and revealed that ATB-346 is useful for the treatment of pain associated with osteoarthritis [150].

These positive preclinical data for ATB-346 suggested that H_2_S-releasing hybrid drugs could undergo further development for the treatment of cardiovascular disease.

Moreover, Martelli and co-workers reported aryl isothiocyanates as another series of thiol-activated H_2_S donors (Figure S12) [57]. These compounds showed vasorelaxant effects on conductance and coronary arteries. 

Noteworthy, 4-carboxyphenyl-isothiocyanate (4-CPI) and 3-pyridyl-isothiocyanate (Figure S20) exhibited cardioprotective effects against I/R injury, reducing myocardial contractility, ventricular arrhythmias, and oxidative stress [58,151].

The mechanism of H_2_S release from these compounds has already been described above (Figure 6). The nature of the R groups supporting the isothiocyanate function have a strong impact on H_2_S-releasing rates. For examples, *p*-nitrophenyl isothiocyanate produces H_2_S relatively fast, while benzylisothiocyanate and phenethyl isothiocyanate are considered slow H_2_S donors [101].

More recently, 1,2,4-thiadiazolidine-3,5-diones (THIA) (Figure S12), which are isothiocyanate derivatives, developed by Severino et al. [53], were demonstrated to be novel thiol-triggered H_2_S donors. 

These compounds were obtained through oxidative condensation of isothiocyanates with isocyanates in the presence of SO_2_Cl_2_, using both the conventional synthetic strategy, previously described in literature [152], and the microwave-assisted synthesis [53]. The second way led better yields being obtained and the reduction of reaction times (Figure S21). The authors clearly defined the mechanism of H_2_S release by using a combination of HPLC, MS, and NMR to isolate and characterize the intermediates and the final products. 

**Figure 12:**
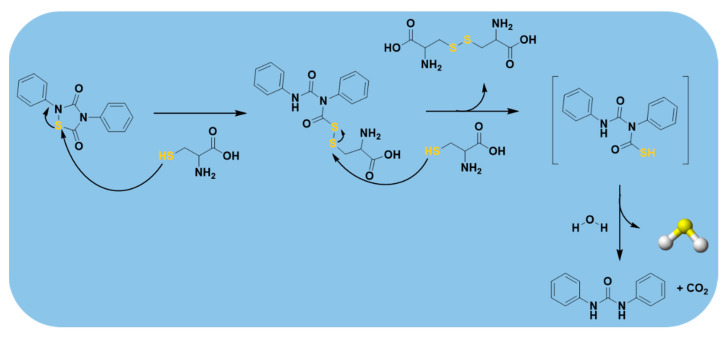
H_2_S-releasing mechanism from 1,2,4-thiadiazolidine-3,5-diones (THIA).

As depicted in Figure 12, the nucleophilic attack of thiols, for example cysteine, on the electron deficient sulfur atom of the 1,2,4-thiadiazolidine-3,5-dione nucleus opens the ring and produces an intermediate. When this latter reacts with a second molecule of cysteine, it generates cystine and phenyl(phenylcarbamoyl)carbamothioic S-acid, an instable intermediate, which is immediately hydrolyzed, yielding 1,3-diphenylurea, CO_2_, and H_2_S. 

It was also found that these donors exerted vasodilating activity in a concentration-dependent manner on isolated aorta rings. 

Furthermore, a sodium polysulthionate, named SG-1002 (Figure S12), was developed. This synthetic H_2_S prodrug is a water-insoluble microcrystalline material, mostly consisting of α-sulfur (about 99% S_8_) hydrophilized with traces of ionic substances, such as sodium sulfate, sodium thiosulfate, and sodium polythionates, which enhance its bioavailability [153]. 

SG-1002 was obtained via comproportionation of sulfur atoms in the -2 and +4 oxidation states in a strongly acidic medium of high ionic strength. This synthetic route, together with properties and some therapeutic applications of SG1002 are described in a patent [154]. H_2_S-releasing from S_8_ is activated by thiols and this reaction with GSH is depicted in Figure S22 [155,156,157].

SG-1002, when administered orally, produced a more sustained and consistent increase in H_2_S and sulfane sulfur levels in models of pressure overload heart failure. Moreover, it improved coronary artery vascular reactivity and attenuated high-fat diet-induced cardiac hypertrophy and dysfunction [153,158,159].

Importantly, SG-1002 has completed the phase I clinical trial where its H_2_S-donating ability in patients with congestive heart failure was investigated [160]. The results of this trial confirmed that SG-1002 is useful both in healthy patients and in patients with heart failure. Indeed, it effectively enhances sulfide and NO levels in patients with heart failure. Nevertheless, further trials are required to test the drug’s efficacy [161]. 

To date, it is the only sulfide-based therapy clinically tested in patients with cardiovascular diseases.

#### 2.3.4. Enzyme-Triggered Donors

In recent years, enzyme-triggered H_2_S donors have been received increasing interest due to their many advantages over the other triggers already mentioned above. Among these, a series of esterase-sensitive prodrugs of H_2_S, based on a lactonization reaction, also known as ‘‘trimethyl lock” (TML) [162], was developed by Zheng et al. [163] (Figure S23).

The first synthesized derivative, HP-101, was obtained starting from compound 1 (Figure S24), which, reacting with Lawesson’s reagent under microwave conditions [164], resulted in an intermediate that was then treated with one equivalent of sodium hydroxide.

**Figure 13:**
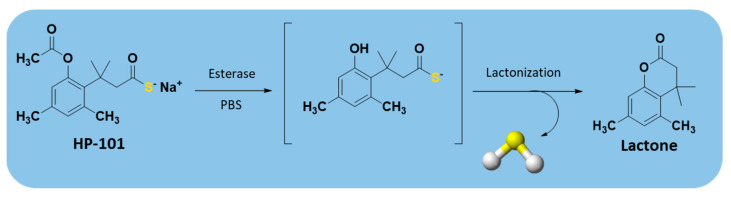
Mechanism of esterase-triggered H_2_S release.

The obtained TML-based prodrugs were able to release H_2_S, through the cleavage of a phenolic ester by an esterase and subsequent lactonization, owing to the steric repulsion of three methyl groups (Figure 13).

Interestingly, a TML derivative, HP-102 (Figure S24), was found to attenuate myocardial I/R injury in a dose-dependent manner and exhibited a cardioprotective action [165]. Thus, this compound is currently under investigation to better define its chemical, pharmacological, and safety profile in the hope that it could be tested in clinical trials.

Another class of enzyme-triggered H_2_S donors is carbonyl sulfide (COS)-releasing compounds. Indeed, COS is a substrate of carbonic anhydrase (CA), which catalyzes its conversion into H_2_S [166,167].

An example of these dual COS/H_2_S donors are N-thiocarboxyanhydrides (NTAs) (Figure S25) [168,169], which have the advantage of releasing, in addition to COS, only innocuous peptide byproducts [170].

The sarcosine NTA derivative (NTA1) (Figure S25) was synthesized starting from sarcosine (N-methyl glycine) through the route depicted in Figure S26 [171].

**Figure 14:**
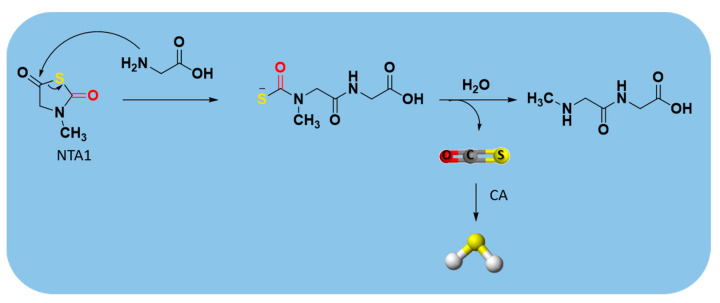
Mechanism of carbonyl sulfide (COS)/H_2_S release from NTA1.

The mechanism leading to H_2_S production from NTA1 consists of ring-opening by a biological nucleophile, such as an amine. The resulting dipeptide byproducts were confirmed by GC-MS and LC-MS, respectively. After COS release, its conversion into H_2_S occurs through the rapid enzymatic action of CA (Figure 14) [170].

Furthermore, Powell et al. showed that the treatment of endothelial cells with NTA1 increased proliferation, which is an important step in the angiogenetic process [170].

Among the class of COS/H_2_S donors, some ROS-triggered H_2_S donors (peroxyTCM-1, 2, 3) were developed [172], using a ROS-cleavable aryl boronate as the protecting group [173,174]. They were obtained as depicted in Figure S27.

**Figure 15:**
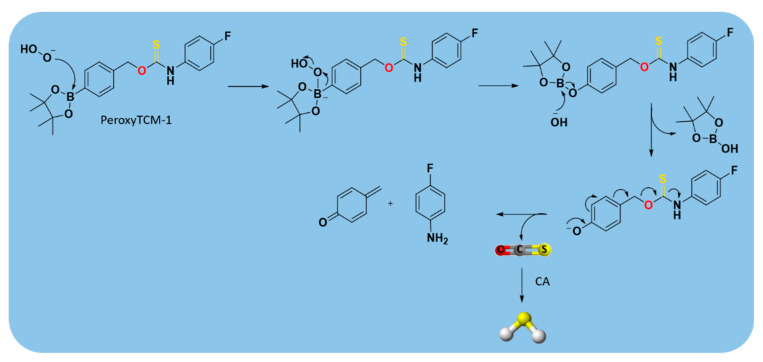
Proposed mechanism for H_2_O_2_-triggered COS/H_2_S release.

The obtained compounds were found to release H_2_S in the presence of an oxidant, such as H_2_O_2_. In this mechanism, the boronate ester was converted to a phenol by ROS, releasing COS, which was quickly converted into H_2_S by CA [172] (Figure 15).

Moreover, it was demonstrated that the combined role of boronate ester, as ROS scavenger, with the produced H_2_S, as cytoprotective agent, led the ROS-activated H_2_S donors to exhibit important activities in many human pathologies driven by high oxidative stress, such as cardiovascular diseases [133]. 

However, further in vivo studies are required to better characterize the biological nature of these donors and to confirm their efficacy.

#### 2.3.5. Others

In 2012, Zhou et al. reported that thioamino acids, such as thioglycine and thiovaline, were bicarbonate-triggered H_2_S donors [48]. They synthesized the selected compounds starting from commercially available Boc-protected amino acids, which were treated with 1,1′-carbonyldiimidazole in dichloromethane and subsequently with gaseous H_2_S. The following deprotection with trifluoroacetic acid (TFA) in dichloromethane furnished the desired compounds (Figure S28). 

**Figure 16:**
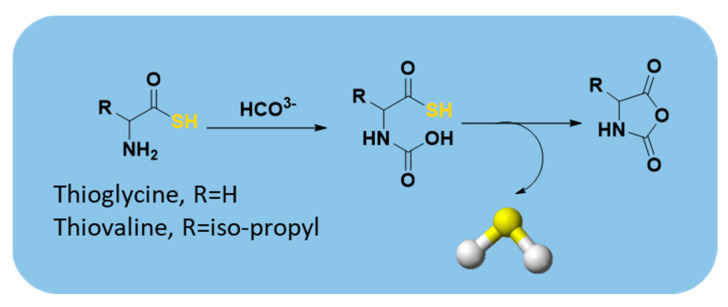
Proposed mechanism for H_2_S release from thioamino acids.

The authors proved that, in the presence of bicarbonate, these compounds react through their amine groups and consequently undergo a cyclization, converting to the corresponding amino acid N-carboxyanhydrides with the production of H_2_S (Figure 16). Considering the proposed mechanism of release and the high concentrations of bicarbonate in blood, thioamino acids could be effective H_2_S donors.

Indeed, H_2_S-releasing capabilities of thioglycine and thiovaline were evaluated and proved amperometrically. In addition, both thioglycine and thiovaline were found to enhance cGMP formation in a concentration-dependent manner and induce significant relaxation of pre-contracted mouse aortic rings. 

However, the high reactivity of these amino acids must be considered. In fact, it was found that under aerobic conditions they could rapidly undergo amidation [175,176,177,178,179,180] and oxidation reactions [51], yielding products which could lead to unwanted side effects. 

Recently, a novel H_2_S-NO-releasing molecule, 2-(N-Boc-amino)-3-prop-2-ynylsulfanylpropionic acid, named ZYZ803, was developed [181] by coupling SPRC with furoxan, upon treatment with Boc anhydride (Figure S29) [181,182].

As already described above, SPRC could enhance H_2_S concentration in plasma through the activation of CSE enzyme [92] while furoxan is a thiol-triggered NO-releasing compound [183].

**Figure 17:**
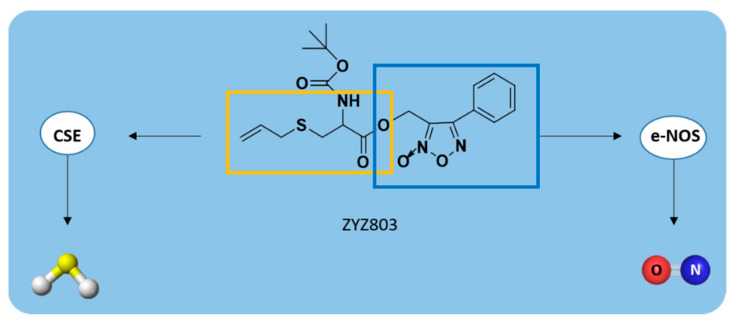
H_2_S/NO-releasing mechanism from ZYZ803.

Therefore, ZYZ803 stimulated both the expression of CSE and the activity of endothelial NO synthase (e-NOS), releasing H_2_S and NO, respectively (Figure 17). These gasotransmitters promoted angiogenesis through the SIRT1/VEGF/cGMP pathway and via cross-talk between STAT3 and CaMKII [184].

As reported by Hu et al., ZYZ803 significantly promoted endothelial cell angiogenesis both in vitro and in vivo [185].

Indeed, the oral administration of ZYZ803 in a mouse model of hindlimb ischemia stimulated angiogenesis more effectively than individually administered SPRC and furoxan, confirming the additive protective actions of H_2_S and NO releasing from ZYZ803 [185].

Additionally, ZYZ803 regulated the vascular tone in isolated rat aortic rings [181], attenuated cardiac dysfunction, and improved myocardial injury after heart failure [186], exerting a powerful cardioprotective effect. 

Lastly, another H_2_S donor with activity in the cardiovascular system is zofenopril, an angiotensin-converting enzyme (ACE) inhibitor. Undergoing hydrolysis in the liver, it forms zofenoprilat, its active metabolite containing a thiol group (Figure S30).

It is important to underline that zofenopril is not a new drug; in fact, it was approved for medical use in 2000. Recently, Bucci et al. demonstrated that this compound can release H_2_S [187], which mediates the beneficial effects of zofenopril, such as anti-inflammatory, pro-angiogenic and anti-apoptotic activity [187,188,189,190,191].

Additionally, zofenopril was reported to improve vascular function in a model of hypertension, combining its ACE inhibitor activity and H_2_S-releasing properties [187].

Indeed, it was also found that zofenopril administration increases the levels of H_2_S metabolites in the plasma of mice, protecting against myocardial I/R injury, and pigs, improving endocardial blood flow in myocardial ischemia [192].

## 3. Conclusions

During last decades, remarkable progress has been made in the understanding of the biological activity of H_2_S and its mechanism of actions in the cardiovascular system and in the discovering and developing of novel H_2_S-releasing compounds.

Herein we presented an overview of the currently available H_2_S donating agents that have been tested in preclinical models of cardiovascular diseases, with a focus on fundamental chemistry and their H_2_S-releasing properties and mechanisms. 

As described above, these H_2_S donors include inorganic sulfide salts, natural and synthetic organic compounds, characterized by different release triggering mechanisms. 

Although many donors have been developed and tested in vitro and in vivo studies, showing their ability to release H_2_S and the positive effects when treating cardiovascular diseases, to date it is not yet possible to identify an ideal donor, owing to some drawbacks for each of them. 

Indeed, the major limitations for many donors are that the release does not mimic the endogenous H_2_S production and leads to the simultaneous generation of reactive byproducts, whose nature and biological activity are often still unknown. 

To overcome these drawbacks, several new classes of donors with a sustained and controlled H_2_S release were designed, synthesized, and studied for their biological activity, also defining their mechanism of release; moreover, they were successfully tested in various animal models of cardiovascular disease. 

Among them, at least two H_2_S-releasing compounds (ATTM and SG-1002) have completed the phase I clinical trials and registered on clinicaltrials.gov for cardiovascular disease and breast cancer (Table 1).

Nevertheless, there are still too few available donors with oral bioavailability, which is crucial for clinical translation, and being available for testing in in vivo pharmacokinetic studies. Furthermore, the safety and long-term effects of many H_2_S-releasing compounds have yet to be more tested and fully characterized.

Bearing in mind the above considerations, this work, by reviewing the available H_2_S donors exhibiting cardioprotective activity and thus providing an insightful knowledge of their chemical nature and mechanisms of release, could enhance the chances to design novel H_2_S-donating molecules useful to help further reduce the risks of cardiovascular disease in the coming years.

## Figures and Tables

**Table 1:** H_2_S-releasing molecules in cardiovascular system.

H_2_S Donors	Chemical Structure	Mechanism of H_2_S Release	Drug Development Phases
**Lawesson’s reagent**	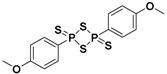	Hydrolysis-triggered	Pharmacological studies
**GYY4137**	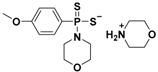	Hydrolysis-triggered	Preclinical trials
**Phosphorodithioates**	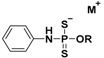	Hydrolysis-triggered	Pharmacological studies
**1,2-dithiole-3-thiones (DTTs)**	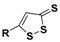	Hydrolysis-triggered	Pharmacological studies
**JK donors**	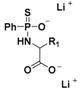	pH-controlled	Preclinical trials
**Ammonium tetrathiomolybdate (ATTM)**	(NH_4_)_2_MoS_4_	pH-controlled	Phase I and II clinical trials for breast cancer
**N-Benzoylthiobenzamides**	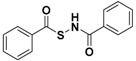	Thiol-triggered	Pharmacological studies
**Acyl perthiols**	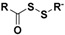	Thiol-triggered	Preclinical trials
**Dithioperoxyanhydrides**	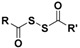	Thiol-triggered	Pharmacological studies
**Arylthioamides**	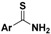	Thiol-triggered	Preclinical trials
**Arylisothiocyanates**	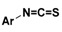	Thiol-triggered	Pharmacological studies
**1,2,4-Thiadiazolidine-3,5-diones**	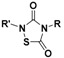	Thiol-triggered	Pharmacological studies
**SG-1002**	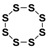	Thiol-triggered	Phase I clinical trial complete for heart failure
**Trimethyl lock (TML)**	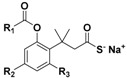	Enzyme-triggered	Pharmacological studies
**N-Thiocarboxyanhydrides** **(NTAs)**	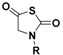	Enzyme-triggered	Preclinical trials
**PeroxyTCM**	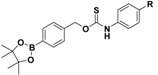	Enzyme-triggered ROS-triggered	Preclinical trials
**Thioamino acids**	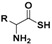	Bicarbonate-triggered	Pharmacological studies
**ZYZ803**	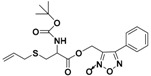	CSE/eNOS-dependent	Preclinical trials
**Zofenopril**	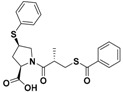	ACE inhibitor	Clinical use for CVD
**ACS 14**	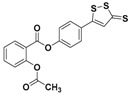	HS–ASA hybrid	Preclinical trials
**AP-39**	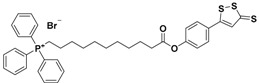	Mithocondrial-targeted	Preclinical trials

## Supplementary Materials

The following are available online at https://www.mdpi.com/2076-3921/10/3/429/s1, The following are available online. Figure S1. The chemical structures of SAC, SPC, and SPRC. Figure S2. Chemical structures of hydrolysis-triggered H_2_S donors. Figure S3. Chemical synthesis of GYY4137. Figure S4. The hydrolytic degradation of GYY4137 and the H_2_S release. Figure S5. Structures of the most used DTTs as H_2_S donors. Figure S6. Different chemical strategies to synthesize DTTs. Figure S7. Mechanism of H_2_S release from DTTs. Figure S8. Chemical synthesis of HS-NSAIDs. Figure S9. Synthetic strategy to obtain JK donors. Figure S10. Chemical structures of two main JK donors. Figure S11. The H_2_S release from ammonium tetrathiomolybdate (ATTM). Figure S12. Chemical structures of thiol-triggered H_2_S donors with cardioprotective activity. Figure S13. Chemical synthesis of N-(benzoylthio)benzamides. Figure S14. Chemical structures of perthiol-based donors. Figure S15. Synthetic route to obtain cysteine perthiol-based donors. Figure S16. Chemical synthesis of penicillamine perthiol-based donors. Figure S17. The synthetic strategies to obtain dithioperoxyanhydride-based H_2_S. Figure S18. Chemical synthesis of (**a**) non-heterocyclic and (**b**) heterocyclic p-hydroxy-arylthio-amides. Figure S19. The thioamides: General structure, chemical structures of lead compound (p-hydroxybenzothioamide) and its hybrid with naproxen (ATB-346). Figure S20. Chemical structures of two aryl isothiocyanates exhibiting cardioprotective activity. Figure S21. Synthesis of THIA. Conventional synthetic strategy: (i) SO_2_Cl_2_/diethyl ether, 24 h; (ii) H_2_O, reflux, 30 min; microwave-assisted synthesis: (i) SO_2_Cl_2_/diethyl ether/MW 500 W, 50 °C, 1h; (ii) H_2_O, MW 500 W, 120 °C, 30 min. Figure S22. Mechanism of H_2_S release from SG1002. Figure S23. Chemical structures of “TML”-based H_2_S prodrugs HP-101 and HP-102. Figure S24. Synthesis of “TML”-based H_2_S prodrug HP-101. Figure S25. Chemical structures of N-thiocarboxyanhydrides (NTAs) and sarcosine NTA derivative (NTA1). Figure S26. Chemical synthesis of sarcosine NTA derivative (NTA1). Figure S27. Chemical structures and synthesis of ROS-activated H_2_S donors. Figure S28. Synthesis of thioamino acids. Figure S29. Chemical structure and synthesis of ZYZ803. Figure S30. Chemical structures of (**a**) zofenopril and (**b**) its active metabolite, zofenoprilat.
