# Supplementary material for: Trends in H2S-Donors Chemistry and Their Effects in Cardiovascular Diseases
Source: Antioxidants (Basel). 2021 Mar 11;10(3):429. doi: 10.3390/antiox10030429 (PMC8002049; doi:10.3390/antiox10030429)
Supplement: Supplementary file 1 [file antioxidants-10-00429-s001.pdf]

SUPPLEMENTARY MATERIALS

Trends in H_2S-donors Chemistry and Their Effects in Cardiovascular Diseases

Angela Corvino, Francesco Frecentese, Elisa Magli, Elisa Perissutti, Vincenzo Santagada, Antonia Scognamiglio, Giuseppe Caliendo, Ferdinando Fiorino, Beatrice Severino*

Department of Pharmacy, School of Medicine, University of Naples «Federico II», Via D. Montesano, 49, 80131, Napoli, Italy; angela.corvino@unina.it (A.C.); frecente@unina.it (F.Fr.); elisa.magli@unina.it (E.M.); perissut@unina.it (E.P.); santagad@unina.it (V.S.); antonia.scognamiglio@unina.it (A.S.); caliendo@unina.it (G.C.); fefiorin@unina.it (F.Fi.)
*Correspondence: bseverin@unina.it (B.S.); Tel. +39081679828

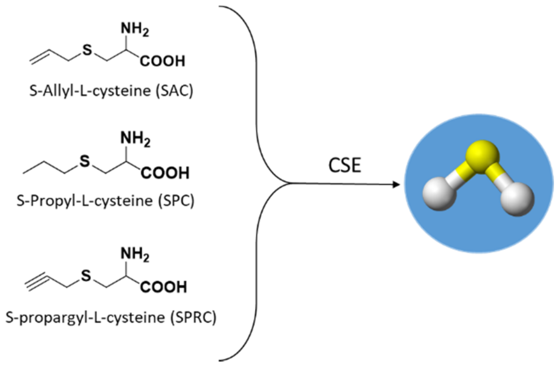

Figure S1. The chemical structures of SAC, SPC and SPRC.

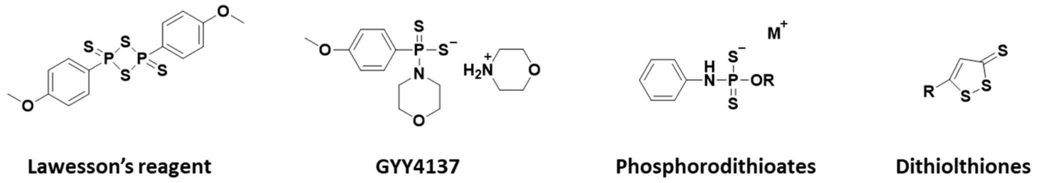

Figure S2. Chemical structures of hydrolysis-triggered H_2S donors.

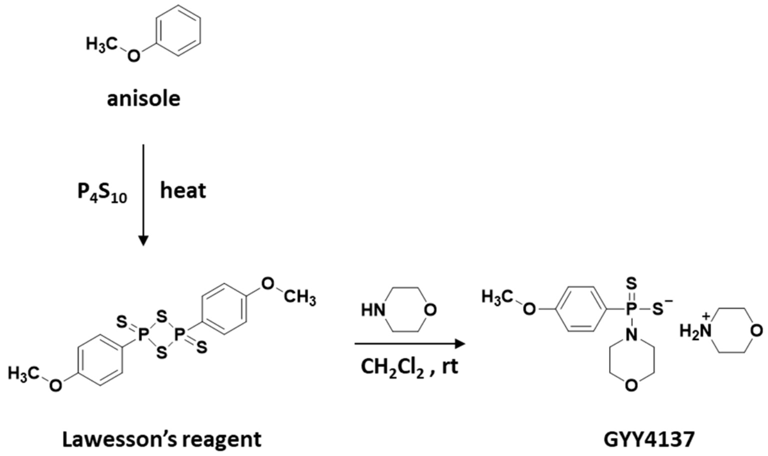

Figure S3. Chemical synthesis of GYY4137.

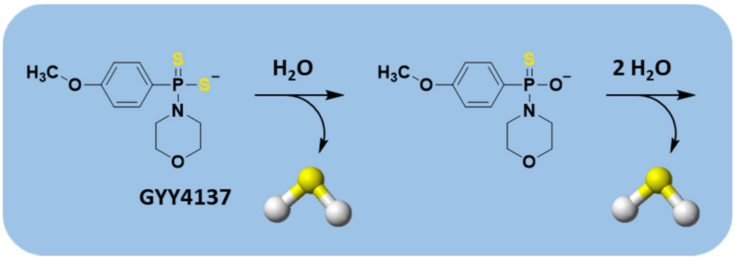

Figure S4. The hydrolytic degradation of GYY4137 and the H_2S release.

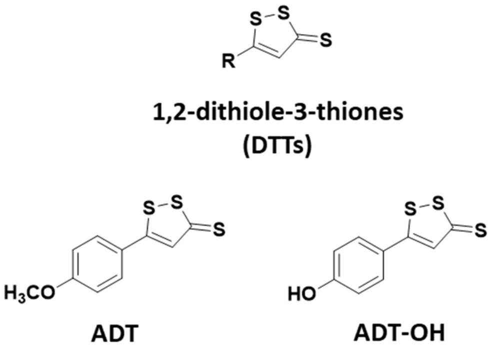

Figure S5. Structures of the most used DTTs as H_2S donors.

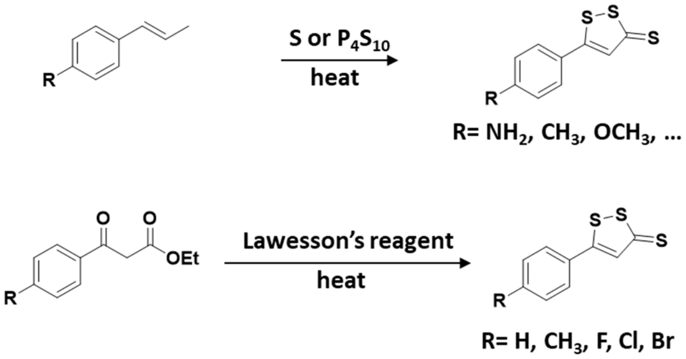

Figure S6. Different chemical strategies to synthesize DTTs.

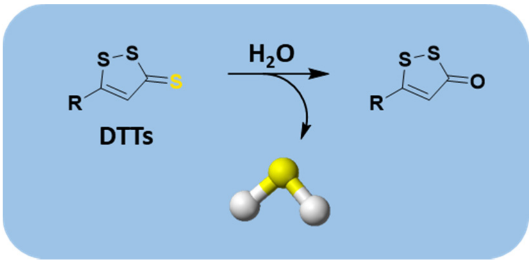

Figure S7. Mechanism of H_2S release from DTTs.

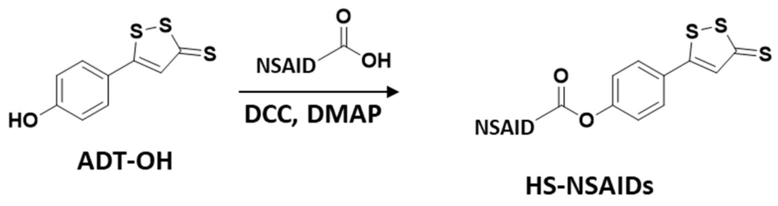

Figure S8. Chemical synthesis of HS-NSAIDs.

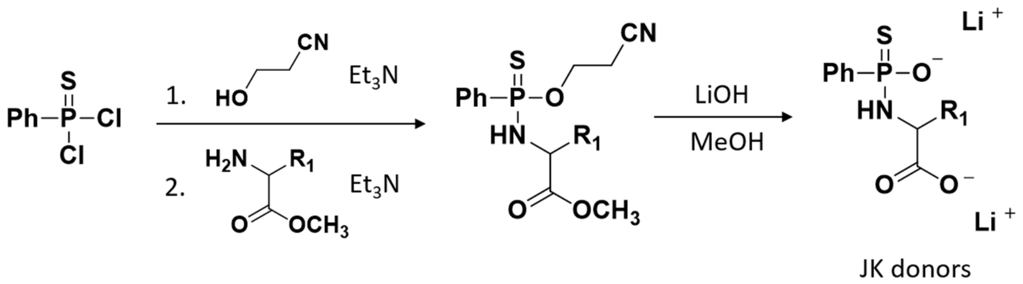

Figure S9. Synthetic strategy to obtain JK donors.

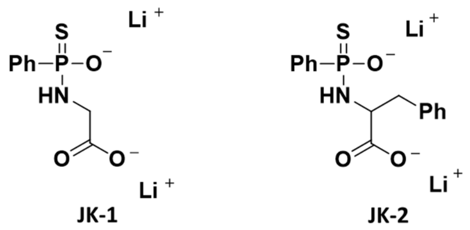

Figure S10. Chemical structures of two main JK donors.

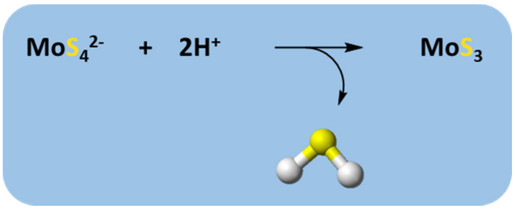

Figure S11. The H_2S release from ammonium tetrathiomolybdate (ATTM).

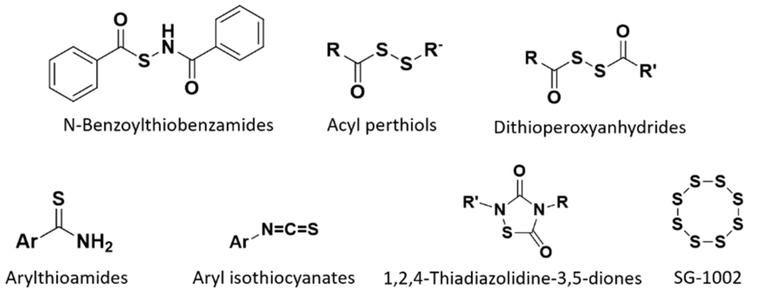

Figure S12. Chemical structures of thiol triggered H_2S donors with cardioprotective activity.

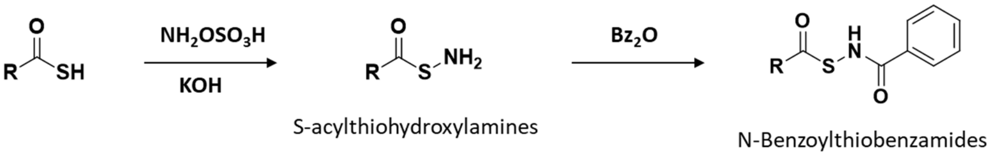

Figure S13. Chemical synthesis of N-(benzoylthio)benzamides.

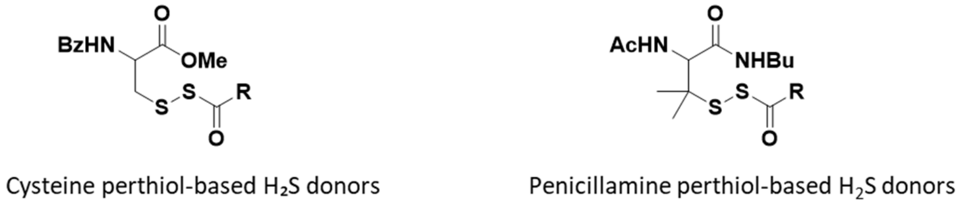

Figure S14. Chemical structures of perthiol-based donors.

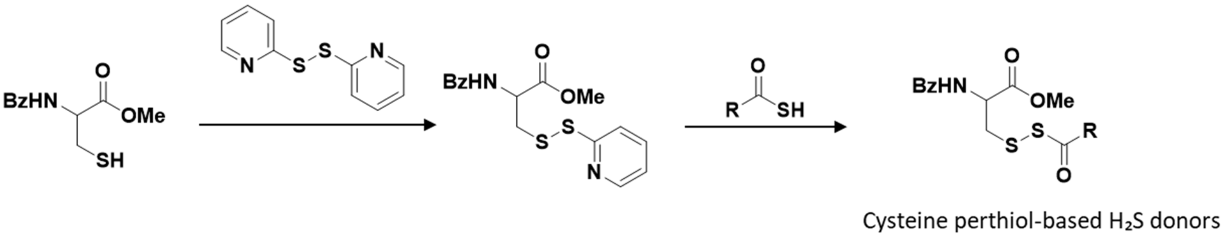

Figure S15. Synthetic route to obtain cysteine perthiol-based donors.

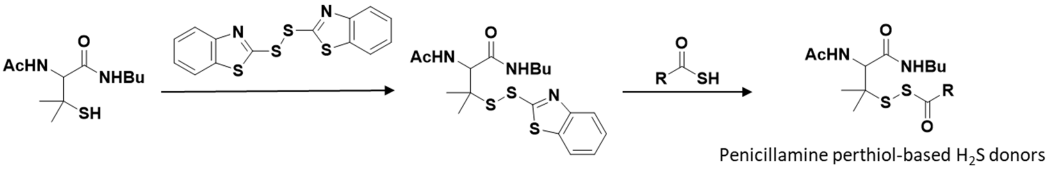

Figure S16. Chemical synthesis of penicillamine perthiol-based donors.

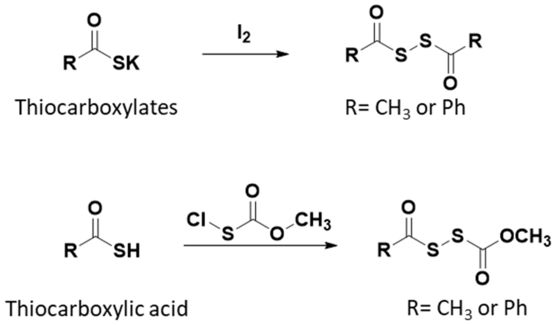

Figure S17. The synthetic strategies to obtain dithioperoxyanhydride-based H_2S**.**

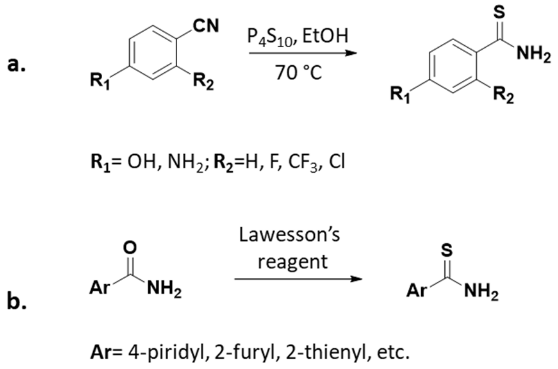

Figure S18. Chemical synthesis of a) non-heterocyclic and b) heterocyclic *p*-hydroxy-arylthio-amides.

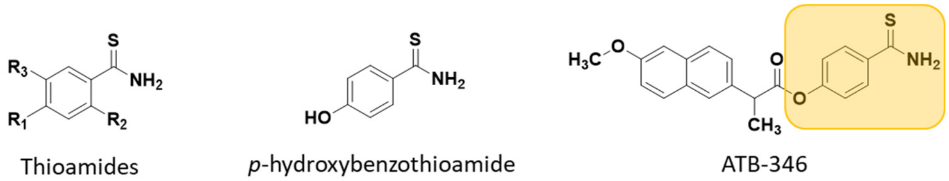

Figure S19. The thioamides: general structure, chemical structures of lead compound (p-hydroxybenzothioamide) and its hybrid with naproxen (ATB-346).

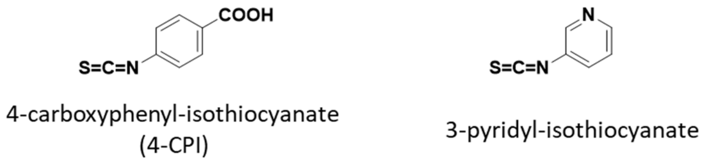

Figure S20. Chemical structures of two aryl isothiocyanates exhibiting cardioprotective activity.

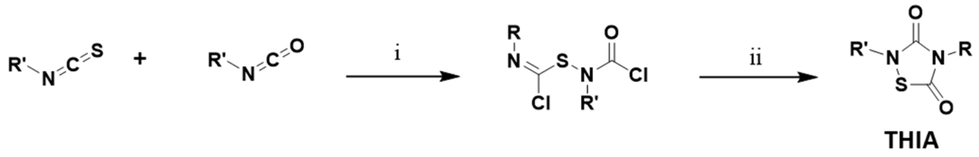

Figure S21. Synthesis of THIA. Conventional synthetic strategy: (i) SO_2Cl_2/diethyl ether, 24h; (ii) H_2O, reflux, 30 min; microwave-assisted synthesis: (i) SO_2Cl_2/diethyl ether/MW 500W, 50 °C, 1h; (ii) H_2O, MW 500W, 120 °C, 30 min.

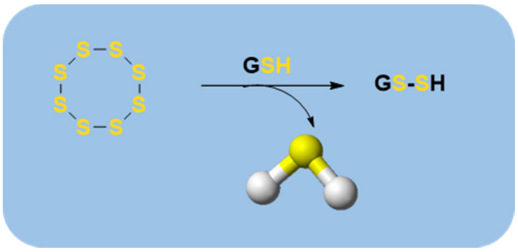

Figure S22. Mechanism of H_2S release from SG1002.

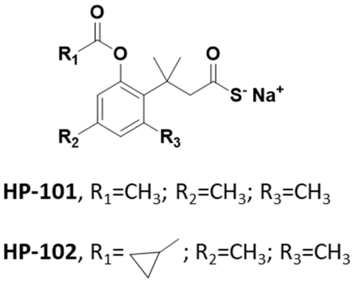

Figure S23. Chemical structures of "TML"-based H_2S prodrugs HP-101 and HP-102.

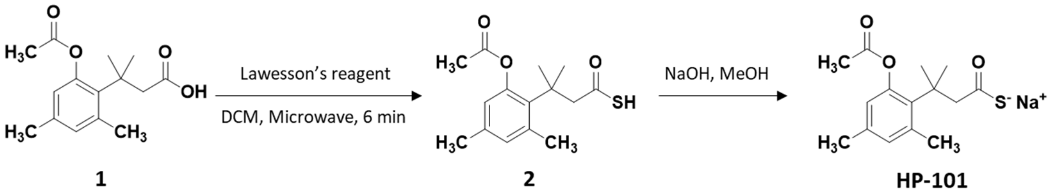

Figure S24. Synthesis of "TML"-based H_2S prodrug HP-101.

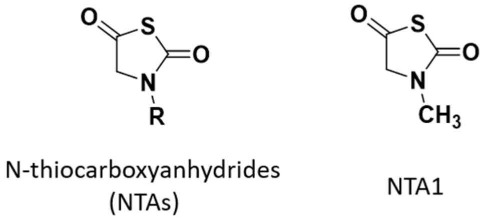

Figure S25. Chemical structures of N-thiocarboxyanhydrides (NTAs) and sarcosine NTA derivative (NTA1).

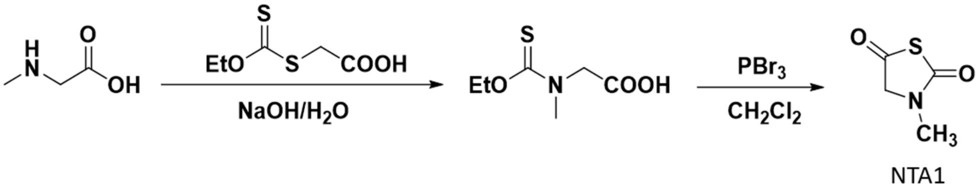

Figure S26. Chemical synthesis of sarcosine NTA derivative (NTA1).

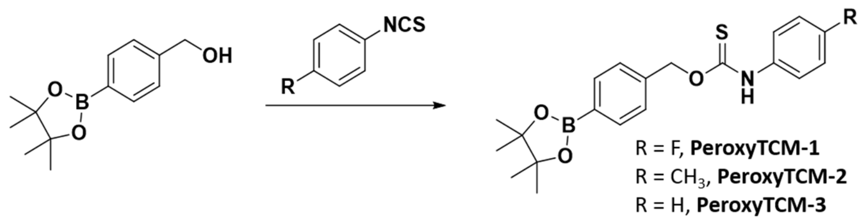

Figure S27. Chemical structures and synthesis of ROS-activated H_2S donors.

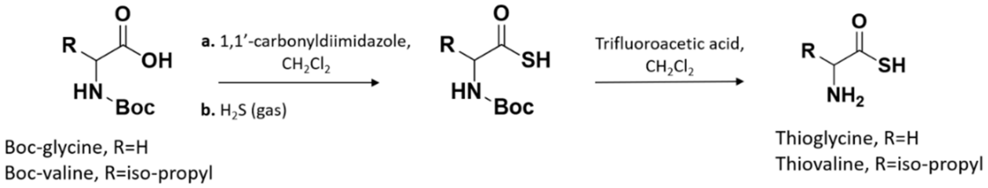

Figure S28. Synthesis of thioamino acids.

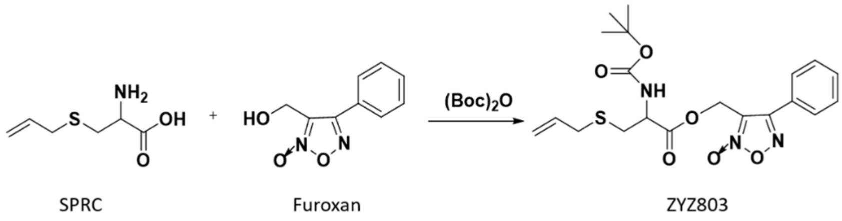

Figure S29. Chemical structure and synthesis of ZYZ803.

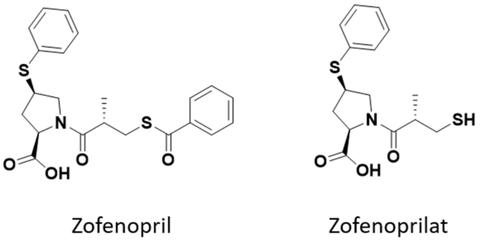

Figure S30. Chemical structures of a) zofenopril and b) its active metabolite, zofenoprilat.
